# *In-Vitro* Evaluation of Two Types of Neonatal Oxygenators in Handling Gaseous Microemboli and Maintaining Optimal Hemodynamic Stability During Cardiopulmonary Bypass

**DOI:** 10.5935/1678-9741.20160075

**Published:** 2016

**Authors:** Neelima Marupudi, Shigang Wang, Luiz Fernando Canêo, Fabio Biscegli Jatene, Allen R. Kunselman, Akif Undar

**Affiliations:** 1 Pediatric Cardiovascular Research Center, Penn State Health Milton Hershey Medical Center, Penn State College of Medicine, Penn State Health Children's Hospital, Hershey, PA, USA.; 2 Instituto do Coração do Hospital das Clínicas da Faculdade de Medicina da Universidade de São Paulo (InCor-HCFMUSP), São Paulo, SP, Brazil.; 3 Public Health Sciences, Penn State Health Milton Hershey Medical Center, Penn State College of Medicine, Penn State Health Children's Hospital, Hershey, PA, USA.; 4 Pediatric Cardiovascular Research Center Surgery and Bioengineering, Penn State Health Milton Hershey Medical Center, Penn State College of Medicine, Penn State Health Children's Hospital, Hershey, PA, USA.

**Keywords:** Cardiopulmonary Bypass. Pediatrics, Oxygenators, Membrane

## Abstract

**Objective:**

Usually only FDA-approved oxygenators are subject of studies by the international scientific community. The objective of this study is to evaluate two types of neonatal membrane oxygenators in terms of transmembrane pressure gradient, hemodynamic energy transmission and gaseous microemboli capture in simulated cardiopulmonary bypass systems.

**Methods:**

We investigated the Braile Infant 1500 (Braile Biomédica, São José do Rio Preto, Brazil), an oxygenator commonly used in Brazilian operating rooms, and compared it to the Dideco Kids D100 (Sorin Group, Arvada, CO, USA), that is an FDA-approved and widely used model in the USA. Cardiopulmonary bypass circuits were primed with lactated Ringer's solution and packed red blood cells (Hematocrit 40%). Trials were conducted at flow rates of 500 ml/min and 700 ml/min at 35ºC and 25ºC. Real-time pressure and flow data were recorded using a custom-based data acquisition system. For gaseous microemboli testing, 5cc of air were manually injected into the venous line. Gaseous microemboli were recorded using the Emboli Detection and Classification Quantifier.

**Results:**

Braile Infant 1500 had a lower pressure drop (*P*<0.01) and a higher total hemodynamic energy delivered to the pseudopatient (*P*<0.01). However, there was a higher raw number of gaseous microemboli seen prior to oxygenator at lower temperatures with the Braile oxygenator compared to the Kids D100 (*P*<0.01).

**Conclusion:**

Braile Infant 1500 oxygenator had a better hemodynamic performance compared to the Dideco Kids D100 oxygenator. Braile had more gaseous microemboli detected at the pre-oxygenator site under hypothermia, but delivered a smaller percentage of air emboli to the pseudopatient than the Dideco oxygenator.

**Table:** 

Abbreviations, acronyms & symbols	
**ANOVA**	**= Analysis of variance**
**CPB**	**= Cardiopulmonary bypass**
**EDAC**	**= Emboli detection and classification**
**EEP**	**= Energy equivalent pressure**
**FDA**	**= Food and Drug Administration**
**GME**	**= Gaseous microemboli**
**THE**	**= Total hemodynamic energy**
**VAVD**	**= Vacuum-assisted venous drainage**

## INTRODUCTION

Brazil, a country of continental dimensions with many regional differences, is experiencing an epidemiological transition, where congenital heart defects and chronic diseases are replacing infections as the primary cause of death^[1]^. Assuming that congenital heart disease can be treated, and that it can be considered a preventable death, the adequate treatment of this population should produce a significant reduction in infant mortality ratio.

Pediatric cardiac surgery is a complex system, where outcomes depend not only on surgical skills, but also on the interaction between human resources, hospitals facilities and processes^[2]^.

Neonates and infants have cardiac lesions with a complex pathophysiology that often require technically demanding procedures, and are prone to complications and/or sequela related to bypass (CPB). Even well-trained and skillful surgeons, while being able to generate excellent results in children, have difficulties reproducing the same kind of outcomes with neonates and infants^[2]^.

Advancements in operative techniques and post-operative care have significantly decreased the mortality of pediatric patients undergoing cardiopulmonary bypass (CPB) procedures in the developed world. However, with this improved survival rate, an increase in morbidity due to surgical and post-surgical complications has been seen^[3]^.

There is significant association between CPB and neurological injury^[4,5]^ due to a variety of mechanisms of neurological insult, such as ischemia, inflammation, and reperfusion injury associated with CPB, which are often exacerbated by problems specific to the pediatric patient because of anatomic, metabolic, and physiological differences compared to the adult population^[5,6]^. Furthermore, the delivery of gaseous microemboli (GME) from the CPB circuit to the patient is believed to be one of the main factors linked to neurological injury^[5,7,8]^. Air may enter the CPB circuitry from a non-occlusive atrial purse string, blood samplings, drug injections, excessive cardiotomy suction return, use of vacuum-assisted venous drainage (VAVD), as well as on initiation of CPB^[9]^. The various components of the CPB circuit, which includes the pump, venous reservoir, cardiotomy reservoir, oxygenator, and arterial filter - when used, also affect the amount of GME delivered to the patient^[10-12]^. Different perfusion methods, flow rates, and temperatures can also have an impact on GME production^[13-15]^. Continuous advancements in the design of CPB products have greatly improved the clearance of GME and thus, clinical outcomes, but constant investigation into safety and efficacy is necessary as companies release new versions of the various CPB components.

Developing a medical industry that could gradually replace imports was a priority from the very beginning of cardiac surgery in some evolving countries like Brazil. Local CPB devices for pediatric patients are now available and approved for clinical use only by the local regulatory health system, without research on its hemodynamics and air-handling capabilities. Therefore, it is not surprising that the large clinical trials published by the international scientific community are generated by testing products approved by the Food and Drug Administration (FDA) and used in developed countries^[16,17]^, and comparing them with other similar devices available in the same region.

Finally, the purpose of this study was to investigate the effectiveness of two neonatal oxygenators: the Braile Infant 1500 (Braile Biomédica, São José do Rio Preto, Brazil), a membrane oxygenator widely used in pediatric CPB procedures in South America though not yet approved by the FDA, and the Dideco KIDS Neonatal D100 (Sorin Group, Arvada, CO, USA), that is FDA-approved and frequently used worldwide. We evaluated and compared the two oxygenators in terms of hemodynamic properties as well as microemboli clearance at both normothermic and hypothermic conditions at varying flow rates and perfusion modes.

## METHODS

### CPB Circuit Design

**Fig. 1:**
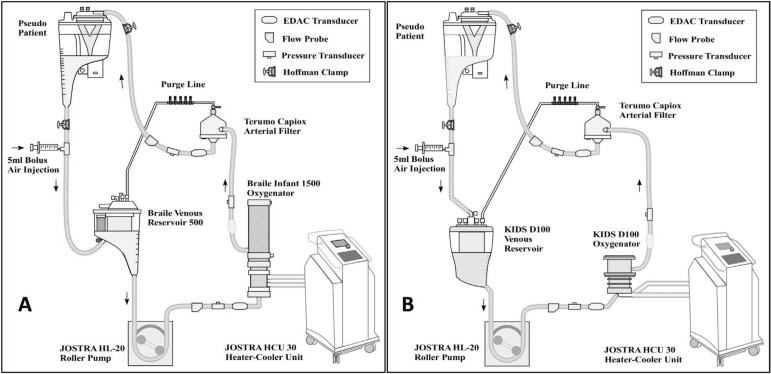
Outline of CPB circuits with the Braile Infant 1500 oxygenator (A) and KIDS D100 oxygenator (B). (In the experimental set-up, the venous reservoir was placed directly on top of the oxygenator. They are separated in this schematic in order to display all components of the circuit clearly).

The experimental circuit was constructed to be identical to the circuit set-up used in the pediatric cardiothoracic operating room. The circuit consisted of an HL-20 roller pump (Jostra, Austin, TX, USA), a Jostra-30 heater-cooler unit (Jostra, Austin, TX, USA), one of the two oxygenators being tested in the experiment and its accompanying venous reservoir, 6 feet of ¼ inch venous tubing, 5 feet of ¼ inch arterial tubing, a custom-made purge line, and a separate Capiox AF02 pediatric arterial filter (Terumo Corporation, Tokyo, Japan) (Figure 1). The two hollow-fiber membrane oxygenators investigated in this study were the Braile Infant 1500 and the Dideco KIDS Neonatal D100 oxygenator. The specifications for each oxygenator and venous reservoir can be seen in Table 1. The purge line consisted of 24 inches of tubing (1/8 in x 1/32 in) connected to a COBE 5 port manifold (Sorin Cardiovascular Inc., Arvada, CO, USA), and then 48 inches of tubing (3/32 in x 1/16 in) connecting the COBE 5 port manifold to the venous reservoir. Thus, the purge line was connected directly to the post-filter de-airing port of the oxygenator and the venous reservoir. A Capiox CR10 hard shell cardiotomy reservoir (Terumo Corporation, Tokyo, Japan) served as a "pseudopatient".

**Table 1:** Oxygenator specifications.

Oxygenator	Braile Infant 1500	KIDS D100
Max Flow Rate	1.5 L/min	700 ml/min
Priming Volume	65 ml	31 ml
Hollow-Fiber Material	Polypropylene	Phosphorylcholine
Bundle Surface Area	0.5 m^2^	0.22 m^2^
Venous reservoir	Braile Venous Reservoir 500	KIDS D100
Capacity	450 ml	500 ml
Cardiotomy Filter Pore Size	200 µm	33 µm
Venous Filter Pore Size	245 µm	51 µm

### Experimental Design

The circuit was primed with Lactated Ringer's solution, first. Then, packed red blood cells were added to the circuit (hematocrit 40%). The total volume of the circuit was 700 ml. The venous reservoir was maintained at 200 ml and the pseudopatient was maintained at 300 ml during the experiments, simulating the average blood volume of a 3-4 kg pediatric patient. In addition, a Hoffman clamp was placed on the arterial line to allow us to maintain a constant arterial pressure of 100 mmHg. Another Hoffman clamp was also placed downstream of the venous reservoir to allow us to balance arterial and venous flow rates and maintain the pseudopatient's volume. The arterial filter purge line was kept open for all experiments.

Five ml of air were injected over 5 seconds into the venous line under both non-pulsatile and pulsatile perfusion conditions, at flow rates of 500 ml/min and 700 ml/min under both normothermic (35ºC) and hypothermic (25ºC) temperatures. A total of 10 air bolus injections were performed at each individual set of conditions for each oxygenator for a total of 160 injections.

### Data Acquisition

Two dual-channel Transonic ultrasound flow probes, model 6XL (Transonic Systems, Inc., Ithaca, NY, USA), and three Maxxim disposable pressure transducers (Maxxim Medical, Inc., Ithaca, NY, USA) were utilized. The flow probes were placed both upstream of the oxygenator and downstream of the arterial filter. The pressure transducers were placed upstream and downstream of the oxygenator as well as downstream of the arterial filter. The pressure transducer and flow meter outputs were connected to a data-acquisition device (NI USB-6521, National Instruments, Austin, TX, USA), and then connected to a computer via USB port. Using the Labview 7.1 software, we obtained a sampling rate of 1000 samples per second, and a 20 second segment of the flow rate and arterial pressure was recorded using the LabView program. The flow rate (f) and pressure (p) values were used to calculate the energy equivalent pressure (EEP) during the time interval between t1 and t2, using the following formula^[18]^:

$$\mathrm{EEP}\;(\mathrm{mmHg})\;=\;\int_{t1}^{t2}\mathrm{fpdt}\;/\;\int_{t1}^{t2}\mathrm{fdt}$$

EEP is a measurement of total hemodynamic energy (THE) per milliliter of blood that passes through a given arterial cross section. THE is then calculated by multiplying the EEP by a conversion factor of 1332.

We used the Emboli Detection and Classification (EDAC) quantifier system (Luna Innovations, Inc., Roanoke, VA, USA) to collect data on the size and number of gaseous microemboli^[19]^. Three transducers were connected to the circuit in the following positions: before the oxygenator, after the oxygenator, and after the arterial filter proximal to the Hoffman clamp. The EDAC system was connected to a computer via USB port, and the data were transferred and analyzed through Microsoft Excel. The EDAC data samples were collected for three minutes after each injection of air. There was a waiting period before proceeding with each one to allow the circuit to clear emboli from the previous injection.

### Pulsatile Perfusion Mode Settings

The pulsatile perfusion setting reproduces the time between two R waves of an electrocardiogram by setting the base flow of the Jostra Roller pump to 20%, the pump head start point to 20%, and the pump head stop point to 80%. The pump head start and stop points represent percentages of one complete pump rotation. A pulsatile pump frequency of 70 beats per minute was used.

### Statistical Analysis

Analysis of variance (ANOVA) models were fit to the continuous outcomes (e.g., pressure drop) to compare both oxygenators (Braile and Dideco) and pulsatile mode (nonpulsatile and pulsatile) at given temperatures (25ºC and 35ºC) and flow rates (500 and 700 ml/min). A general linear model with correlated errors was fit to the continuous hemodynamic outcomes (*e.g.,* THE) to compare oxygenators, pulsatile modes, and location in the circuit (*e.g*., pre-oxygenator, post-filter) within given temperatures and flow rates^[20]^. The general linear model with correlated errors is an extension of linear regression that accounts for the within-subject variability inherent to repeated measures designs. In this study, the repeated factor is the location in the circuit. For each outcome, *P*-values were adjusted for multiple comparisons testing using the Tukey procedure. All hypotheses tests were two-sided and all analyses were performed using SAS software, version 9.4 (SAS Institute Inc., Cary, NC, USA).

## RESULTS

### Gaseous Microemboli

Total GME counts delivered to the pseudopatient increased with increasing flow rates and decreasing blood temperatures (Tables 2 and 3). At hypothermic temperature, the Braile oxygenator had more GME recorded at the pre-oxygenator site compared to the Dideco oxygenator (*P*<0.01). At normothermic temperature and 700 ml/min flow rate, the Dideco oxygenator had a higher number of GME at the pre-oxygenator site (*P*<0.01). The majority of microemboli were smaller than 40 µm during all trials. There was no statistically significant difference between the two oxygenators at the post-oxygenator site and distal arterial line. When comparing GME delivered to the pseudopatient as a percentage of GME generated prior to oxygenator (post-oxygenator GME divided by pre-oxygenator GME times 100), the Braile oxygenator delivered a smaller percentage to the pseudopatient than the Dideco oxygenator (*P*<0.01) (Tables 2 and 3).

**Table 2:** GME volumes and counts at 35ºC.

Flow rate	Oxygenator	Mode	Pre-oxygenator site			Post-oxygenator site			Distal arterial line			% GME Count
			Volume (CC)	Count (n)	>40µm (n)	Volume (CC)	Count (n)	>40µm (n)	Volume (CC)	Count (n)	>40µm (n)	
500ml/min	Braile	NP	7.1E-07±4.8E-07	70±30	4±3	1.6E-07±2.8E-07	9±9	1±2	1.5E-09±2.0E-09	1±2	0	13.2
		P	8.5E-07±5.9E-07	108±41	5±5	1.6E-07±2.3E-07	8±6	1±1	1.5E-07±4.4E-07	3±4	0	7.1
	Dideco	NP	1.3E-07±9.3E-08	18±12	1±1	6.9E-08±1.1E-07	6±6	0	1.1E-09±3.1E-09	0±1	0	33.9*
		P	1.6E-07±1.2E-07	19±10	1±1	1.6E-08±2.0E-08	5±5	0	1.0E-10±3.2E-10	0±0	0	26.3*
700 ml/min	Braile	NP	8.7E-07±1.1E-07	115±14	6±1	3.6E-07±6.1E-07	11±5	1±1	8.9E-09±1.6E-08	2±2	0	9.7
		P	1.5E-06±5.5E-07	191±52	7±4	3.7E-07±5.0E-07	16±11	2±3	2.5E-09±1.9E-09	2±1	0	8.5
	Dideco	NP	1.5E-06±6.1E-07	236±98*	5±3	1.1E-06±1.0E-06	99±45	6±7	3.0E-08±2.4E-08	16±9	0	42.1*
		P	1.5E-06±5.6E-07	256±51	5±4	2.6E-06±6.8E-06	107±87	9±21	3.1E-08±2.1E-08	13±7	0	41.8*

NP=non-pulsatile flow; P=pulsatile flow % GME Count=Post-oxygenator count/Pre-oxygenator count x 100;

* *P*<0.01, Braile *vs*. Dideco

**Table 3:** GME volumes and counts at 25ºC.

Flow rate	Oxygenator	Mode	Pre-oxygenator site			Post-oxygenator site			Distal arterial line			% GME Count
			Volume (CC)	Count (n)	>40µm (n)	Volume (CC)	Count (n)	>40µm (n)	Volume (CC)	Count (n)	>40µm (n)	
500 ml/min	Braile	NP	1.0E-06±6.3E-07	164±31	5±4	4.6E-08±7.9E-08	7±6	0±1	1.7E-09±2.7E-09	1±2	0	4.6
		P	1.2E-06±3.6E-07	244±32	5±3	3.1E-08±3.0E-08	9±5	0	1.4E-08±3.4E-08	1±3	0	3.7
	Dideco	NP	1.2E-07±1.1E-07	24±12*	1±1	1.9E-08±1.9E-08	6±4	0	4.1E-10±9.5E-10	1±2	0	25.4*
		P	1.4E-07±7.6E-08	40±8*	1±1	2.3E-08±3.8E-08	6±6	0	1.3E-10±2.7E-10	0±0.5	0	14.3
700 ml/min	Braile	NP	5.1E-06±3.4E-06	654±242	27±21	5.6E-07±7.9E-07	87±60	3±5	9.7E-08±1.0E-07	26±22	0	13.2
		P	8.1E-06±1.2E-06†	1322±203†	39±8†	6.3E-07±5.1E-07	120±22	1±2	8.8E-08±5.6E-08	24±10	0±1	9.1
	Dideco	NP	1.1E-06±4.1E-07*	184±55*	5±3*	4.4E-07±2.0E-07	87±33	2±1	4.3E-08±3.9E-08	16±8	0	47.3*
		P	2.0E-06±7.8E-07*	256±36*	9±4*	9.3E-07±3.7E-07	121±28	4±3	5.8E-08±3.4E-08	25±8	0	47.4*

NP=non-pulsatile flow; *P*=pulsatile flow % GME Count=Post-oxygenator count/Pre-oxygenator count x 100;

* *P*<0.01, Braile *vs*. Dideco;

† *P*<0.05, NP vs. P mode

### Pressure Drop and Hemodynamic Energy

For both oxygenators, the mean pressure drop across the oxygenator increased with a higher flow rate at both temperatures (Table 4). Consistently, the pressure drop was slightly lower at a higher temperature for both oxygenators. The Braile oxygenator showed a lower mean pressure drop than the Dideco (*P*<0.01). This difference was particularly highlighted at a higher flow rate.

**Table 4:** Pressure drop across the oxygenators and “Stolen” blood flow from arterial filter purge line.

Flow rate	Oxygenator	Mode	Oxygenator pressure drop (mmHg)		Stolen blood flow (ml/min)	
			35ºC	25ºC	35ºC	25ºC
500 ml/min	Braile	NP	35.3±0.3	39.8±0.2	132.8±2.9	138.2±0.8
		P	36.4±0.3	41.1±0.3	134.7±3.6	138.5±1.8
	Dideco	NP	92.2±2.3*	110.7±0.8*	128.9±4.3	122.9±0.5*
		P	94.4±1.7*	112.9±1.3*	129.8±5.3	122.9±1.3*
700 ml/min	Braile	NP	47.5±0.1	54.3±0.0	154.3±1.5	135.8±1.1
		P	49.4±0.3	56.3±0.3	155.4±1.7	136.7±1.7
	Dideco	NP	125.5±0.2*	163.7±2.9*	133.1±0.4*	115.6±1.4*
		P	129.8±0.7*†	168.0±2.9 *†	133.9±1.8*	116.1±2.2*

NP=non-pulsatile flow; P=pulsatile flow

* *P*<0.01, Braile *vs*. Dideco;

† *P*<0.05, NP *vs*. P mode

**Fig. 2:**
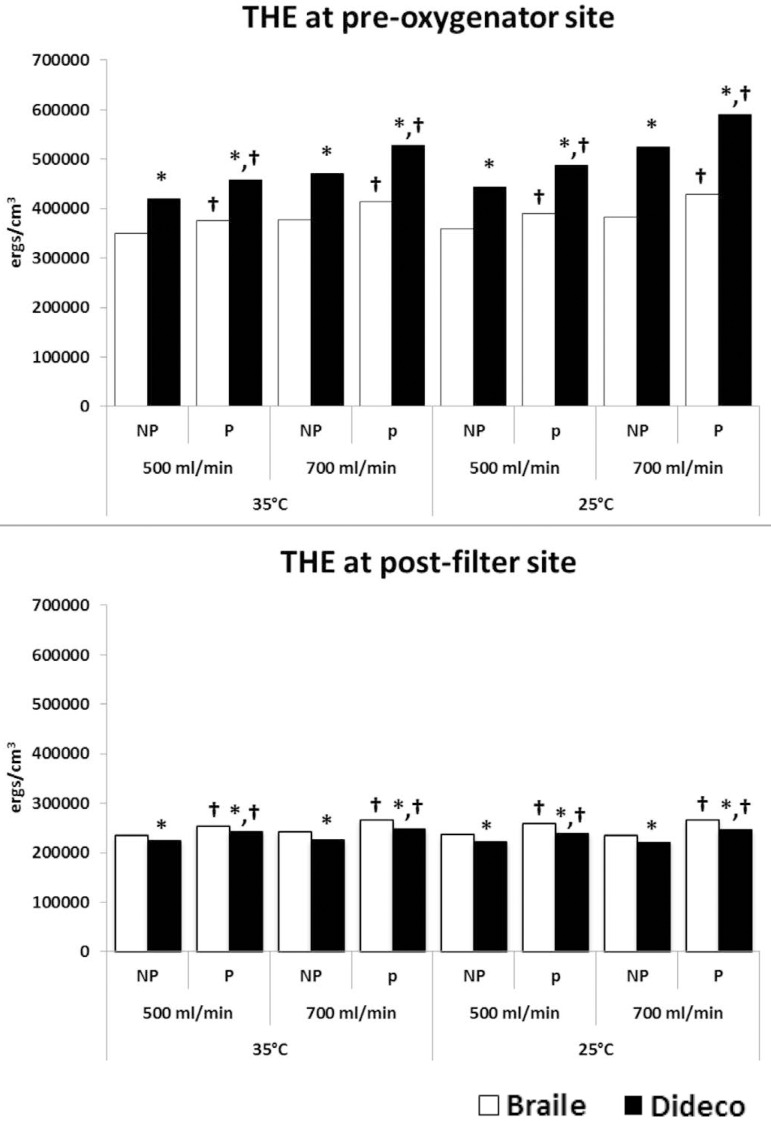
Pre-oxygenator and post-filter total hemodynamic energy (THE) under non-pulsatile (NP) and pulsatile (P) mode. *P<0.01, Braile vs. Dideco; †P<0.01, NP vs. P mode

**Fig. 3:**
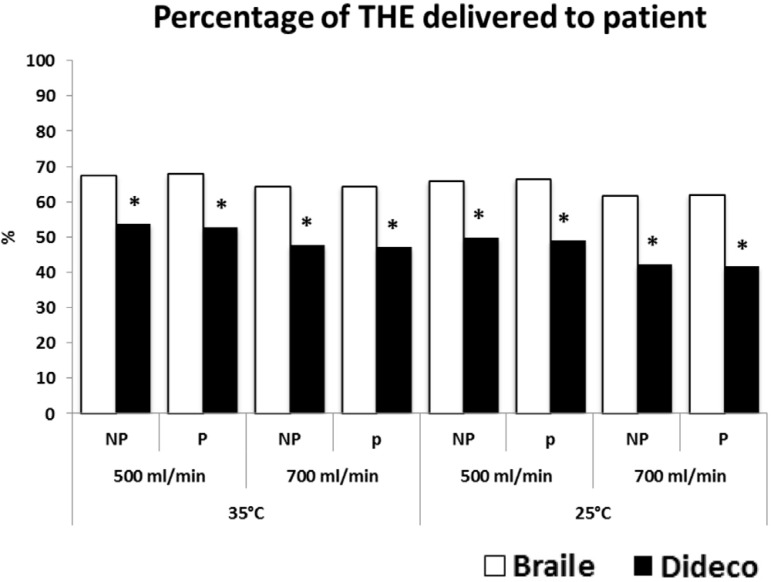
Percentage of pre-oxygenator total hemodynamic energy (THE) delivered to pseudopatient under non-pulsatile (NP) and pulsatile (P) mode. *P<0.01, Braile vs. Dideco

THE decreased across the oxygenator in all experimental conditions. Pre-oxygenator THEs were higher at a higher flow rate as well as in hypothermic conditions (Figure 2). The Dideco oxygenator had higher pre-oxygenator THE than the Braile in all experimental conditions, with a lower post-filter THE delivered to the pseudopatient (*P*<0.01). The Dideco oxygenator exhibited a greater drop in THE across the oxygenator, resulting in a smaller percentage of original post-oxygenator THE being delivered to the patient as compared to the Braile oxygenator (*P*<0.01) (Figure 3).

### "Stolen" Blood Flow

Blood flow shunted through the purge line of the arterial filter from the patient increased at higher flow rates and, in general, also increased at normothermia. The Braile oxygenator had a higher rate of "stolen" blood flow from the pseudopatient at a higher flow rate and hypothermia (*P*<0.01) (Table 4).

### Pulsatile and Non-Pulsatile Perfusion Modes

There was a slightly higher pressure drop as well as stolen blood flow at the pulsatile condition for both oxygenators (Table 4). The oxygenator pressure drop reached statistical difference (*P*<0.05) between non-pulsatile and pulsatile modes only at 700 ml/min in the Dideco group. The pre-oxygenator and post-arterial filter THEs were higher at pulsatile conditions (*P*<0.01) (Figure 2). However, the percentage of pre-oxygenator THE delivered to the patient was not significantly different between the two perfusion modes (Figure 3). There was always a higher number of GME generated prior to oxygenator under pulsatile mode as compared to the non-pulsatile mode, but there was a significant difference only at the pre-oxygenator site at 25ºC in the Braile group (*P*<0.05). In addition, the percentage of oxygenator GME trapping was similar between the two perfusion modes (*P*>0.05) (Tables 2 and 3).

## DISCUSSION

Gaseous microemboli remain an important challenge in CPB procedures because of the significant positive correlation between microemboli exposure during CPB and postoperative neurological injury^[21]^. Thus, minimizing the number of GME delivered to pediatric patients undergoing CPB procedures should lead to better clinical outcomes. Sources of air emboli are numerous and include bubbles in the venous line, the vent and suction lines, vacuum assisted venous drainage, perfusionist handling, as well as manual manipulation by the surgeon^[9]^. Most GME should be removed by the oxygenator, which is why we continue to test the various oxygenators on the market to determine which ones are the most effective at this task. In the present study, we found that the number of microemboli detected prior to oxygenator and following oxygenator was larger at higher flow rates. We believe that this happens because higher flow rates decrease the time the blood spends across the venous filters, thus preventing optimal microemboli trapping. In addition, total GME counts slightly increased under pulsatile mode compared to non-pulsatile mode, although there is no statistically significant difference between the two perfusion modes. This may be explained by reducing GME removal at a high instant flow rate under pulsatile mode. We also found the number of GME to be increased in hypothermic conditions due to increased blood viscosity. Therefore, flow rate, blood temperature and perfusion mode have great effects on GME transmission during CPB procedure, confirming our previous findings^[13-15,22-25]^.

The total GME count was higher for the Braile oxygenator before oxygenator at hypothermia and a higher flow rate. Discrepancies in GME produced before the oxygenator can be attributed to the differences in venous reservoir construction, capacity and filter pore size. The Braile venous reservoir's maximum capacity is 450 ml and the venous filter pore size was 245 micrometers whereas the Dideco KIDS venous reservoir had a maximum capacity of 500 ml and a venous filter pore size of 51 micrometers. The smaller filter size could have played a role in the number of emboli delivered to the pseudopatient. Both limiting the number of microemboli delivered to the patient and maintaining optimal hemodynamic properties are important factors in determining the efficacy of the components of a CPB circuit to reduce morbidity and mortality, particularly linked to neurological damage, after open heart surgery. Regarding the post-oxygenator microemboli as a percentage of the preoxygenator, the Braile oxygenator appears to capture a greater percentage of microemboli because of the discrepancies in the venous filter port sizes and the membrane surface area.

The pressure drop across the oxygenators is specific to the hollow-fiber configuration of each type of oxygenator. This may be in part due to the differences in membrane surface area, maximum blood flow, and fiber density of each oxygenator. The membrane surface area of the Braile oxygenator is 0.5 m^2^, more than double the size of the Dideco oxygenator, which is 0.22 m^2^. Maximum flow rate for the Braile oxygenator (1.5 L/min) was also more than double that of the Dideco oxygenator (700 ml/min). The pressure drop was significantly higher across the Dideco oxygenator than across the Braile at all flow rates, in both pulsatile and non-pulsatile modes. Those differences are important because a higher resistance of the circuit flow leads to a higher pressure drop across the oxygenator, meaning that the blood is being pushed only at a higher pressure against the membranes while passing through the oxygenator. This force is a potential cause for cellular damage and an increased inflammatory response that may significantly delay post-operative recovery. THE is a function of pump flow rate and arterial pressure; thus, the higher pressure drop seen with the Dideco oxygenator could explain why we see much higher pre-oxygenator THE values and lower post-oxygenator THE values, leading to a significant decrease in THE delivered to the patient with this oxygenator when compared to the Braile oxygenator. Pulsatile flow generates significantly greater THE than non-pulsatile flow regardless of type of oxygenator and blood temperature.

Another major factor for neurological injury is the amount of blood shunted through an open arterial purge line. It has been shown that keeping the arterial purge line open can further reduce the total volume and size of microemboli delivered to the patient^[22-25]^. However, keeping the purge line open also shunts a significant amount of blood away from the patient and puts the patient at risk for hypoperfusion and a decreased post-arterial filter THE, especially at lower flow rates^[26]^. We should measure the true flow rate of blood to the patient using flow probes after the arterial filter. These circumstances are all parameters that can be affected by the types of devices used, the flow rate settings, and the temperature and viscosity of the blood. In addition, higher flow rates also result in a higher pressure drop across the oxygenator and a lower percentage of THE delivered to the patient. Thus, the ideal circuit would consist of an arterial filter and oxygenator that limit this "stolen" blood flow and pressure drop across the oxygenator while restricting the volume and size of microemboli delivered to the patient.

### Limitations

The most significant limitation of this study was that the maximum flow rate of each oxygenator was vastly different. The Braile oxygenator had a flow rate of 1.5 L/min whereas the Dideco oxygenator had a flow rate of 700 ml/min. The differing flow rates may influence the pressure gradient across the oxygenator, thus affecting resistance and potential for retained post-oxygenator GME. Although these differences are important, we feel that the oxygenators can and should be compared in terms of efficacy because they are used in the same types of medical procedures for the same patient population.

## CONCLUSION

Our results showed that the Braile Infant 1500 had a lower pressure drop and a higher total hemodynamic energy delivered to the pseudopatient in our simulated pediatric CPB circuits. There was a higher raw number of microemboli detected with the Braile Infant 1500 oxygenator at pre-oxygenator site in hypothermic conditions compared to the Dideco KIDS D100. However, the Braile oxygenator delivered a smaller percentage of micoemboli to the pseudopatient than the Dideco oxygenator. The higher number of GME could be attributed to the varying sizes of their respective venous reservoirs capacity, screen filters, and maximum flow rates. The greater capability of the Braile oxygenator to capture a greater percentage of microemboli could be explained by the discrepancies in the venous filter port size and the membrane surface area between both oxygenators. Hypothermia, pulsatile conditions and higher flow rates tended to deliver a higher number of GME compared to non-pulsatile conditions. Further studies are warranted to verify our findings.

**Table:** 

Authors’ roles & responsibilities	
NM	Analysis and/or data interpretation; conception and design study; manuscript redaction or critical review of its content; realization of operations and/or trials; statistical analysis; final manuscript approval
SW	Analysis and/or data interpretation; conception and design study; manuscript redaction or critical review of its content; realization of operations and/or trials; statistical analysis; final manuscript approval
LFC	Analysis and/or data interpretation; conception and design study; manuscript redaction or critical review of its content; realization of operations and/or trials; statistical analysis; final manuscript approval
FBJ	Analysis and/or data interpretation; conception and design study; manuscript redaction or critical review of its content; realization of operations and/or trials; statistical analysis; final manuscript approval
ARK	Analysis and/or data interpretation; conception and design study; manuscript redaction or critical review of its content; realization of operations and/or trials; statistical analysis; final manuscript approval
AU	Analysis and/or data interpretation; conception and design study; manuscript redaction or critical review of its content; realization of operations and/or trials; statistical analysis; final manuscript approval
